# Assessment of quality of work life (QWL) among healthcare staff of intensive care unit (ICU) and emergency unit during COVID-19 outbreak using WHOQoL-BREF

**DOI:** 10.1016/j.jsps.2021.09.002

**Published:** 2021-09-20

**Authors:** Muhammad Bilal Maqsood, Md. Ashraful Islam, Zeb-un- Nisa, Atta Abbas Naqvi, Ali Al Qarni, Aseel Fuad Al-karasneh, Wajiha Iffat, Syed Azizullah Ghori, Azfar Ather Ishaqui, Akram Hasan Aljaffan, Saleh Alghamdi, Mohammad Aref Albanghali, Ahmad Jamal Mahrous, Muhammad Shahid Iqbal, Amer Hayat Khan, Abdul Haseeb

**Affiliations:** a King Abdullah International Medical Research Center- Eastern Region, Al Ahsa, Saudia Arabia; b King Saud bin AbdulAziz University for Health Sciences, Ministry of National Guard Health Affairs, Al Ahsa, Saudi Arabia; c Swiss Business School, Kloten-Zurich, Switzerland; d Department of Pharmacy Practice, College of Clinical Pharmacy, Imam Abdulrahman Bin Faisal, University, Dammam, Saudi Arabia; e Faculty of Pharmacy, Ziauddin University, Karachi, Pakistan; f Department of Medicine, Ministry of National Guard Health Affairs, AlAhsa, Saudi Arabia; g Dow College of Pharmacy, Dow University of Health Sciences, Karachi, Pakistan; h Department of Pharmacy, King Abdul Aziz Hospital, Ministry of National Guard Health Affairs, AlAhsa, Saudi Arabia; i Discipline of Clinical Pharmacy, School of Pharmaceutical Sciences, Universiti Sains Malaysia, Penang, Malaysia; j Dubai Health Authority, Dubai, United Arab Emirates; k Department of Clinical Pharmacy, Faculty of Clinical Pharmacy, Albaha University, Al baha, Saudi Arabia; l Department of Public Health, Faculty of Applied Medical Sciences, Albaha University, Al baha, Saudi Arabia; m Department of Clinical Pharmacy, College of Pharmacy, Umm Al Qura University, Makkah, Saudi Arabia; n Department of Clinical Pharmacy, College of Pharmacy, Prince Sattam bin Abdulaziz University, Alkharj, Saudi Arabia

**Keywords:** Quality of work life (QWL), Health personnel, Health services, COVID-19, Saudi Arabia

## Abstract

**Objective:**

The study aimed to document the quality of work life (QWL) among healthcare staff of intensive care units (ICUs) and emergency units during COVID-19 outbreak using the WHOQoL-BREF.

**Methods:**

A multicenter cross-sectional study was conducted for two months (May – June 2020) among healthcare staff working in intensive care units (ICUs) and emergency units of the hospitals under the National Guard Health Authority (NGHA) across five cities of Saudi Arabia. The study used the WHOQoL-BREF instrument to document the QWL through an electronic institutional survey. The data was analyzed through IBM SPSS version 23. The study was approved by an ethics committee.

**Results:**

A total of 290 healthcare professionals responded to the survey. The mean overall quality of life score was 3.37 ± 0.97, general health = 3.66 ± 0.88, domains, i.e., physical = 11.67 ± 2.16, psychological = 13.08 ± 2.14, social = 13.22 ± 3.31 and environment = 12.38 ± 2.59. Respondents aged > 40 years, male gender, married status, being a physician and, having a work experience > 15 years and no extra working hours, had higher mean scores for several domains of Quality of life (QoL), overall QoL and general health (p < 0.05).

**Conclusion:**

The QWL among healthcare staff during COVID-19 pandemic was low. Demographic factors were mainly the determinants for a higher QWL while the variable of extra working hours was a determinant of lower QWL. Despite the pandemic, no COVID-19 related variables affected the work life of healthcare staff.

## 1 Introduction

Quality of work life (QWL) could be defined as the way an employee considers and/or evaluates the work in the context of his/her life (Van Laar et al., 2007). QWL could be further elaborated as an individual’s life that may be affected by work (Sulaiman et al., 2015). Van Laar et al. mentioned that QWL of workforce is an important aspect for the employers (Van Laar et al., 2007). High levels of QWL among employees may lead to employment satisfaction and better work engagement (Sulaiman et al., 2015, Sinval et al., 2020). This may further lead to effective staff retention in an organization and may attract prospective staff in future (Mosadeghrad, 2013).

Literature mentions that health profession is quite satisfying and at the same time challenging (Kumar et al., 2018). Healthcare staff working in the hospitals are subjected to established pressure in this profession (Kumar et al., 2018, Liu et al., 2020). While healthcare professionals (HCPs) may feel satisfied in treating patients, they are subjected to high level of stress due to work-load, nature of work, higher risk of exposure (Kumar et al., 2018). Additionally HCPs involved in treating COVID-19 patients may have stress owing to the work practice setting and, lack of expertise in infectious disease, etc. (Liu et al., 2020, Iffat et al., 2021). Available evidence mentions that in most countries, nurses experience high levels of work stress (Lambert & Lambert, 2001). This stress and burnout could lead to a lower quality of life at work (Kumar et al., 2018). This is important to document and address since a lower QWL among healthcare staff could compromise the quality of patient care. In a study among nurses in Australia, the majority of nurse mentioned their inability to meet the needs of their patients due to staffing and funding issues, and reasoned it as a cause of frustration and burnout (Hegney et al., 2003). It was observed in a study that depressed residents were six times more likely to make medications errors (Fahrenkopf et al., 2008, O’Hagan and Richards, 1998). The advent of COVID-19 pandemic further increased the pressure on hospital staff since HCPs were the first ones to come in contact with SARS-CoV-2 (Alserehi et al., 2020). There have been reports of COVID-19 related suicide cases among HCPs worldwide (Jahan et al., 2021).

In Saudi Arabia, several studies have been conducted to report the work-related quality of life among nursing staff (Almalki et al., 2012, Alharbi et al., 2019). Alharbi and colleagues reported a moderate quality of work life among nurses in Madinah region (Alharbi et al., 2019). Another study among nurses in Southern region highlighted a high level of job dissatisfaction (Almalki et al., 2012). Both studies highlighted several factors that acted as determinants of QoL (Almalki et al., 2012, Alharbi et al., 2019). Literature reports that HCPs may experience extra pressure and stress while caring for patients with COVID-19 (Abolfatouh et al., 2020). With an increasing number of cases, more beds were occupied with patients. This increased the workload of the healthcare staff and at the same time, posed a threat of exposure to the virus. Hence, it was important to know the quality of work life QWL of HCPs during these unprecedented times. The study aimed to document the work-related quality of life among healthcare staff of intensive care unit (ICU) and emergency unit during COVID-19 outbreak.

## 2 Methods

### 2.1 Study design, venue, and duration

This was a multicenter cross-sectional study and was conducted for two months (May – June 2020) in intensive care units (ICUs) and emergency units of the hospitals under the National Guard Health Authority (NGHA) in Riyadh, Jeddah, Medina, Dammam and Alahsa, Saudi Arabia. Riyadh is located in the central region of the country. It is the capital of Saudi Arabia and the most populated city. Jeddah, Medina, Dammam, are capitals of their respective provinces. Jeddah is the second most populous port city in the western region. Madinah is also a located in the western region 4th most populated city. Dammam and Alahsa are located in the eastern region. Dammam is also a port city and 6th most populated city (World Population Review, 2021).

### 2.2 Participants and eligibility criteria

All healthcare professionals namely physicians, pharmacists, nurse, and allied health team members, who were licensed to practice, working in intensive care units (ICUs) and emergency units, and for at least 1 year, were invited to participate in this study. The healthcare staff who did not have at least 1 year of work experience, and those who did not agree to participate in the study were excluded. The reason to not include staff with < 1 year of work experience was that such personnel may not have experienced the work environment sufficiently enough to be able to distinguish their QWL before and during the pandemic.

### 2.3 Sampling strategy and sample size

Probability sampling technique was adopted, and an online survey link was emailed to all healthcare staff of the healthcare facilities under the Ministry of National Guard Health Affairs (MNGHA) (MNGHA, 2021). It was carried out through institutional communication. The email network of the institution was linked to all healthcare facilities all over Saudi Arabia. The request for survey was made to the office responsible for communication, by submitting the questionnaire and a copy of ethics approval. The communication office sent the survey through the institutional emails to all employees. The screening criteria, i.e., ‘frontline healthcare workers’, was explicitly mentioned in the email to encourage only relevant healthcare professionals to respond.

The sample size was calculated by an online calculator (Raosoft, 2021). According to the McKinsey Global Institute Report of 2015, there were about 350,000 HCPs working in the country (McKinsey Global Institute, 2015). This figure was identified as the target population. For the current study, minimum number of 267 samples were required considering 95% confidence level and 6% marginal error. Due to nature of online data collection technique used, additional sample were enrolled to compensate for potential missing or unintended error (Sakpal, 2010). The adjusted sample size formula is:

$$n_{1}=n/\left(1-e\right)$$

where n is required sample size as per formula, n_1_ is adjusted sample size and, e is the potential missing or unintended error of the samples. Considering 10% potential missing or unintended error of the samples, the adjusted sample size was 296.67. Thus, the sample size targeted for this study was 300 from different health care professionals.

### 2.4 Research instrument

The WHOQoL-BREF was used with authorization from the World Health Organization (WHO) (permission authorization ID: 325823) on 28th March 2020. (WHO, 2020). The scale contains 26 items related to the different aspects of quality of life. The scale provides a score in four different domains of QoL namely physical, psychological, social, and environmental. In addition, it also provides a score for overall QoL and general health (WHO, 1996).

### 2.5 Data analysis

The data was collected using the online survey link in the form of MS Excel spreadsheet that was imported and analyzed using IBM SPSS version 23 (Armonk, USA). Informal technique was used to trace out the missing cases. Some of them (n = 7) were treated using ‘last-observation-carried-forward method’, technique that is commonly used in pharmaceutical research, and few cases (n = 10) were excluded from final analysis (Lang, 2007). Discrete data was expressed in mean (X), Range (R), and standard deviation (SD) while categorical data was reported in number (N) and frequency (%). The inferential statistics included independent sample *t*-test that was used to evaluate the mean difference in regards to the participants’ background characteristics in all four health domains of QoL, overall QoL and general health.

Additionally, the hierarchical regression analysis was utilized to evaluate the predictors of QoL in all four health domains, overall QoL and general health. The significant variables of age, gender and social status were adjusted in the first model. Similarly, significant variables related to the work-conditions namely occupation and work experience were adjusted in the second model. In the third model, all significant COVID-19 related variables, i.e., any extra working hours, caring for patient with COVID-19 infections were adjusted. Multicollinearity were checked using VIF and tolerance value and no multicollinearity was found. The models were checked for linearity, homoscedasticity, normality of residuals and autocorrelation of residual. Statistical significance was accepted at p < 0.05.

### 2.6 Consent and ethics approval

All participants were provided with an electronic written informed consent before the actual survey. They were required to provide their consent. After checking the consent checkbox, they were directed to the electronic survey form. The study was subjected to an ethics review by Institutional Review Board of King Abdullah International Medical Research Center and was approved on 15th April 2020, (RA20/012/A).

## 3 Results

Of 300 anticipated responses, a total sample of 290 healthcare professionals were analyzed for this study giving a response rate of 96%. Most participants were female (N = 185, 63.8%), married (N = 191, 65.9%), had bachelor’s degree (N = 225, 77.6%) and were between 31 and 40 years (N = 123, 42.4%). The majority of healthcare professionals were non-Saudi (N = 208, 71.7%) and worked in hospitals of Riyadh region (N = 93, 32.1%). Most healthcare professionals belonged to the profession of nursing (N = 179, 61.7%) and had a work experience between 10 and 15 years (85, 29.3%). More than a third of healthcare professionals (N = 112, 38.6%) were directly involved in caring for patients with COVID-19 and did not have any extra working hours (N = 96, 33.1%). The background characteristics and QoL score are summarized in Table 1, Table 2, respectively.

**Table 1:** Background characteristics (N = 290).

Characteristics	Frequency	Percent
**Age**		
20–30 years	54	18.6
31–40 years	123	42.4
41–50 years	104	35.9
51–60 years	2	0.7
>60 years	7	2.4
**Gender**		
Male	105	36.2
Female	185	63.8
**Social Status**		
Single	91	31.4
Married	191	65.9
Divorced	7	2.4
Widowed	1	0.3
**Educational qualification**		
Bachelors	225	77.6
Masters	36	12.4
Doctorate	29	10
**Nationality**		
Saudi	82	28.3
Non-Saudi	208	71.7
**Occupation**		
Doctor	51	17.6
Pharmacist	6	2.1
Nurse	179	61.7
Allied Health	54	18.6
**Region**		
Riyadh	93	32.1
Jeddah	82	28.3
Alahsa	53	18.3
Dammam	39	13.4
Medina	23	7.9
**Work Experience**		
Between 1 and 5 years	60	20.7
Between 5 and 10 years	56	19.3
Between 10 and 15 years	85	29.3
Between 15 and 20 years	44	15.2
> 20 years	45	15.5
**Involved in direct care of patients with COVID – 19**		
No	178	61.4
Yes	112	38.6
**Extra working hour**		
No extra working hours	96	33.1
≤18 h (1–2 extra shift)	79	27.2
> 18 ≤ 36 h (3–4 extra shift)	83	28.6
> 36 ≤ 54 h (5–6 extra shift)	21	7.2
> 54 h (>7 extra shift)	11	3.8

**Table 2:** Summary of the quality of life scores from WHOQoL-BREF domains.

Quality of life domains	Mean (SD)	Range (Min-Max)	Cronbach's alpha	Standardized Cronbach's alpha
Overall QoL (score out of 5)	3.37 (0.97)	1.00–5.00	---	---
General Health (score out of 5)	3.66 (0.88)	1.00–5.00	---	---
Physical	11.67 (2.16)	5.14–17.14	0.578	0.601
Psychological	13.08 (2.14)	6.67–17.33	0.570	0.578
Social	13.22 (3.31)	5.33–20.00	0.704	0.712
Environment	12.38 (2.59)	4.50–19.00	0.811	0.815

Bivariate analysis revealed a significant mean difference in all four health domains, overall QoL and general health for several demographic variables. A significant (p < 0.001) difference in mean score for the variable of age was observed in Physical, Psychological, Social, and general health. Further, significant difference at p < 0.05 and p < 0.01 in mean score was observed for the variable of age in Environment and overall Qol respectively. Healthcare professionals who were > 40 years of age had higher mean scores. There was a significant difference (p < 0.05) in mean score for the variable of gender in Psychological and Environment domains as well as general health. The male staff had higher mean scores. Similarly, there was a significant difference (p < 0.05) in mean score for the variable of social status in Physical, Social and Environment domains as well as general health. It was significant at p < 0.01 for Social domain. Those who indicated their social status as married had higher mean scores for the said domains and general health. For the variable of occupation, there was a significant difference (p < 0.05) in Environment domain as healthcare professionals who were physicians had higher mean score as opposed to all other HCPs when grouped together as non-physicians.

Furthermore, there was a significant difference (p < 0.05) in mean score for the variable of work experience in Physical, Psychological and Social domains as well as general health. It was significant at p < 0.01 for Social domain. The healthcare staff having work experience of more than 15 years had higher mean scores. In addition, there was a significant difference (p < 0.05) in mean score for the variable of extra working hours in Physical and Environment domains as well as overall QoL and general health. The staff who had no extra working hours had higher mean scores. No significant difference (p > 0.05) was observed in variable of education and having direct contact with a patient with COVID-19 (Table 3).

**Table 3:** Bivariate analysis highlighting mean differences in scores for health domains.

Variables	Health domains					
	**Physical**	**Psychological**	**Social**	**Environment**	**Overall QoL**	**General Health**
	**Mean (SD)**	**Mean (SD)**	**Mean (SD)**	**Mean (SD)**	**Mean (SD)**	**Mean (SD)**
**Age (p-value)**	<0.001	<0.001	<0.001	0.042	0.002	<0.001
≤40 years	11.29 (2.21)	12.66 (2.12)	12.66 (3.39)	12.14 (2.59)	3.23 (0.98)	3.48 (0.87)
>40 years	12.27 (1.93)	13.73 (2.01)	14.11 (3.00)	12.77 (2.55)	3.58 (0.91)	3.93 (0.83)
**Gender (p-value)**	0.230	0.035	0.937	0.026	0.925	0.049
Male	11.87 (2.18)	13.43 (2.21)	13.23 (3.41)	12.83 (2.58)	3.36 (1.05)	3.79 (0.93)
Female	11.56 (2.14)	12.88 (2.08)	13.22 (3.27)	12.13 (2.57)	3.37 (0.92)	3.58 (0.85)
**Social status (p-value)**	0.044	0.089	0.001	0.048	0.108	0.024
Married	11.85 (2.05)	13.23 (2.12)	13.68 (3.18)	12.59 (2.49)	3.43 (0.94)	3.74 (0.83)
Single	11.32 (2.31)	12.78 (2.16)	12.35 (3.41)	11.96 (2.75)	3.24 (1.00)	3.48 (0.96)
**Education (p-value)**	0.532	0.900	0.927	0.397	0.384	0.827
Bachelors	11.72 (2.14)	13.07 (2.13)	13.23 (3.31)	12.31 (2.55)	3.40 (0.94)	3.66 (0.83)
Others (Masters & Doctorate)	11.53 (2.20)	13.11 (2.17)	13.19 (3.34)	12.62 (2.75)	3.28 (1.05)	3.63 (1.07)
**Occupation 1 (p-value)**	0.483	0.280	0.912	0.046	0.772	0.251
Physician	11.87 (2.24)	13.37 (2.11)	13.18 (3.40)	13.04 (2.56)	3.33 (1.11)	3.78 (0.97)
Others (Allied health + Nurse + Pharmacist)	11.63 (2.14)	13.02 (2.14)	13.23 (3.30)	12.24 (2.58)	3.38 (0.94)	3.63 (0.84)
**Occupation 2 (p-value)**	0.988	0.102	0.268	0.209	0.622	0.392
Nurse	11.68 (2.12)	12.92 (2.06)	13.39 (3.25)	12.23 (2.52)	3.39 (0.92)	3.62 (0.81)
Others (Allied health + Physician + Pharmacist)	11.67 (2.22)	13.34 (2.24)	12.95 (3.41)	12.63 (2.69)	3.33 (1.04)	3.71 (0.98)
**Occupation 3 (p-value)**	0.443	0.525	0.582	0.122	0.644	0.345
Allied health	11.87 (2.22)	13.24 (2.24)	13.01 (3.42)	12.86 (2.62)	3.32 (1.09)	3.75 (0.97)
Others (Nurse + Physician + Pharmacist)	11.62 (2.14)	13.04 (2.12)	13.28 (3.29)	12.27 (2.58)	3.38 (0.93)	3.63 (0.86)
**Work experience (p-value)**	0.033	0.015	0.006	0.074	0.109	<0.001
≤15 years	11.49 (2.21)	12.88 (2.10)	12.87 (3.26)	12.20 (2.51)	3.31 (0.98)	3.52 (0.90)
>15 years	12.08 (1.98)	13.54 (2.16)	14.02 (3.32)	12.79 (2.74)	3.51 (0.92)	3.97 (0.76)
**Extra working hours (p-value)**	0.045	0.142	0.161	0.012	0.032	0.010
No	12.02 (11.50)	13.34 (2.25)	13.61 (3.09)	12.93 (2.59)	3.54 (0.99)	3.84 (0.89)
Yes	11.50 (2.09)	12.95 (2.07)	13.03 (3.41)	12.11 (2.55)	3.28 (0.94)	3.56 (0.86)
**Direct contact with COVID-19 patients (p-value)**	0.370	0.739	0.351	0.785	0.479	0.622
No	11.58 (2.13)	13.04 (2.14)	13.08 (3.34)	12.42 (2.53)	3.34 (1.00)	3.63 (0.92)
Yes	11.82 (2.20)	13.13 (2.15)	13.45 (3.28)	12.33 (2.69)	3.42 (0.91)	3.69 (0.83)
*Applied Independent sample t test*						

In addition, the model for Physical domain revealed that with an increasing age above 40 years the mean score would increase by 0.237 when variables related to the socio-demographic and work condition are considered. Moreover, it would increase by 0.222 when all variables are considered (p < 0.001). No significant predictor was reported among variables related to work condition and COVID-19 (Table 4).

**Table 4:** Predictors of physical domain of QoL.

Factors	Model 1 Coefficient (p-value)	Model 2 Coefficient (p-value)	Model 3 Coefficient (p-value)
**Socio-demographic variables**			
**Age in years** (0, ≤ 40; 1, > 40)	0.209 (<0.001)	0.237 (0.002)	0.222 (0.004)
**Marital status** (0, Married; 1, Single)	−0.082 (0.158)	−0.085 (0.145)	−0.083 (0.158)
**Work condition variable**			
**Work experience** (0, ≤15 years; 1, >15 years)		−0.044 (0.563)	−0.040 (0.603)
**COVID-19 related variables**			
**Extra working hours** (0, No; 1, Yes)			−0.079 (0.178)
**Direct contact with COVID-19 patients** (0, No; 1, Yes)			0.034 (0.558)
**Regression** (F (df), p-value)	8.557 (2), p < 0.001	5.803 (3), p = 0.001	3.947 (5), p = 0.002
R2	0.056	0.057	0.065
R2 change	0.056	0.001	0.008

The model for Psychological domain highlighted that with an increasing age above 40 years, the mean score would increase by 0.275 if variables related to the socio-demographic and work condition are considered. It would increase by 0.270 if all variables are accounted for (p < 0.01). In addition, the model predicted that the mean score would decrease by 0.137 if variables related to the socio-demographic and work condition are considered. It would decrease by 0.134 if all variables are accounted for (p < 0.05). No significant predictor was reported among variables related to work condition and COVID-19 (Table 5).

**Table 5:** Predictors of psychological domain of QoL.

Factors	Model 1 Coefficient (p-value)	Model 2 Coefficient (p-value)	Model 3 Coefficient (p-value)
**Socio-demographic variables**			
**Age in years** (0, ≤ 40; 1, > 40)	0.249 (<0.001)	0.275 (<0.001)	0.270 (<0.001)
**Gender** (0, Male; 1, Female)	−0.134 (0.019)	−0.137 (0.017)	−0.134 (0.021)
**Work condition variable**			
**Work experience** (0, ≤ 15 years; 1, > 15 years)		−0.040 (0.597)	−0.041 (0.589)
**COVID-19 related variables**			
**Extra working hour** (0, No; 1, Yes)			−0.038 (0.514)
**Direct contact with COVID-19 patients** (0, No; 1, Yes)			0.003 (0.955)
**Regression** (F (df), p-value)	12.015 (2), p < 0.001	8.084 (3), p < 0.001	4.909 (5), p < 0.001
R2	0.077	0.078	0.080
R2 change	0.077	0.001	0.002

The model for Social domain of QoL revealed that for an increasing age of HCP, the mean score would increase by 0.175 if variables related to socio-demographics and work condition are considered. It would increase by 0.164 if all variables are accounted for (p < 0.05). The model also revealed that the mean score would decrease by 0.156 if the HCPs are single provided variables related to socio-demographics and work conditions are considered. It would decrease by 0.153 for the same if all variables are kept under consideration (p < 0.01). No significant predictor was reported among variables related to work condition and COVID-19 (Table 6).

**Table 6:** Predictors of social domain of QoL.

Factors	Model 1 Coefficient (p-value)	Model 2 Coefficient (p-value)	Model 3 Coefficient (p-value)
**Socio-demographic variables**			
**Age in years** (0, ≤ 40; 1, > 40)	0.188 (0.001)	0.175 (0.020)	0.164 (0.031)
**Marital status** (0, Married; 1, Single)	−0.158 (0.007)	−0.156 (0.008)	−0.153 (0.009)
**Work condition variable**			
**Work experience** (0, ≤ 15 years; 1, > 15 years)		0.019 (0.802)	0.025 (0.746)
**COVID-19 related variables**			
**Extra working hours** (0, No; 1, Yes)			−0.049 (0.402)
**Direct contact with COVID-19 patients** (0, No; 1, Yes)			0.039 (0.506)
**Regression** (F (df), p-value)	10.830 (2), p < 0.001	7.217 (3), p < 0.001	4.568 (5), p = 0.001
R2	0.070	0.070	0.074
R2 change	0.070	0.000	0.004

The model revealed that the mean score would increase by 0.125 for an increasing age of HCPs above 40 years (p < 0.05) provided socio-demographic variables are considered. Similarly for the variable of gender, the mean score would decrease by 0.137 for female HCP (p < 0.05) provided socio-demographic variables are considered. Moreover, the model revealed that the mean score would decrease by 0.121 if the healthcare professional worked in extra shifts when all variables related to socio-demographic, work condition and COVID-19 are considered (p < 0.05). No significant predictor was reported among variables related to work condition (Table 7).

**Table 7:** Predictors of environmental domain of QoL.

Factors	Model 1 Coefficient (p-value)	Model 2 Coefficient (p-value)	Model 3 Coefficient (p-value)
**Socio-demographics variables**			
**Age in years** (0, ≤ 40; 1, > 40)	0.125 (0.032)	0.100 (0.191)	0.088 (0.256)
**Gender** (0, Male; 1, Female)	−0.137 (0.019)	−0.104 (0.114)	−0.100 (0.130)
**Work condition variables**			
**Occupation** (0, Physician; 1, Others)		−0.065 (0.321)	−0.063 (0.335)
**Work experience** (0, ≤ 15 years; 1, > 15 years)		0.034 (0.656)	0.024 (0.756)
**COVID-19 related variables**			
**Extra working hours** (0, No; 1, Yes)			−0.121 (0.041)
**Direct contact with COVID-19 patients** (0, No; 1, Yes)			−0.042 (0.483)
**Regression** (F (df), p-value)	4.879 (2), p = 0.008	2.732 (4), p = 0.029	2.594 (6), p = 0.018
R2	0.033	0.037	0.052
R2 change	0.033	0.004	0.015

The model revealed that with an increasing age above 40 years, the mean score would increase by 0.202 if variables related to the socio-demographic and work condition are considered (p < 0.01). It would increase by 0.185 if all variables are accounted for (p < 0.05). No significant predictor was reported among variables related to work condition and COVID-19 (Table 8).

**Table 8:** Predictors of overall QoL.

Factors	Model 1 Coefficient (p-value)	Model 2 Coefficient (p-value)	Model 3 Coefficient (p-value)
**Socio-demographic variable**			
**Age in years** (0, ≤ 40; 1, > 40)	0.178 (0.002)	0.202 (0.008)	0.185 (0.017)
**Work condition variable**			
**Work experience** (0, ≤ 15 years; 1, > 15 years)		−0.037 (0.629)	−0.034 (0.655)
**COVID-19 related variables**			
**Extra working hours** (0, No; 1, Yes)			−0.099 (0.095)
**Direct contact with COVID-19 patients** (0, No; 1, Yes)			0.030 (0.608)
**Regression** (F (df), p-value)	9.454 (1), p = 0.002	4.389 (2), p = 0.009	3.230 (4), p = 0.013
R2	0.032	0.033	0.043
R2 change	0.032	0.001	0.011

The model revealed that with an increasing age above 40 years, the mean score would increase by 0.241 if variables related to the socio-demographic are considered (p < 0.001). It would increase by 0.171 if variable related to both socio-demographic and work condition are considered (p < 0.05). It would increase by 0.153 if all variables are accounted for (p < 0.05). No significant predictor was reported among variables related to work condition and COVID-19 (Table 9).

**Table 9:** Predictors of general health.

Factors	Model 1 Coefficient (p-value)	Model 2 Coefficient (p-value)	Model 3 Coefficient (p-value)
**Socio-demographic variables**			
**Age in years** (0, ≤ 40; 1, > 40)	0.241 (<0.001)	0.171 (0.024)	0.153 (0.044)
**Gender** (0, Male; 1, Female)	−0.107 (0.070)	−0.103 (0.082)	−0.091 (0.125)
**Marital status** (0, Married; 1, Single)	−0.068 (0.255)	−0.062 (0.302)	−0.066 (0.276)
**Work condition variable**			
**Work experience** (0, ≤ 15 years; 1, > 15 years)		0.110 (0.142)	0.111 (0.144)
**COVID-19 related variables**			
**Extra working hours** (0, No; 1, Yes)			−0.104 (0.073)
**Direct contact with COVID-19 patients** (0, No; 1, Yes)			0.015 (0.791)
**Regression** (F (df), p-value)	8.492 (3), p < 0.001	6.939 (4), p < 0.001	5.215 (6), p < 0.001
R2	0.082	0.089	0.100
R2 change	0.082	0.007	0.011

## 4 Discussion

This multi-center study was conducted in one of the largest healthcare facilities of Saudi Arabia during COVID-19 pandemic. There was a significant difference in mean score for the variable of age in all domains, i.e., Physical, Psychological, Social, Environment as well as overall QoL and general health as healthcare professionals (HCPs) who were > 40 years of age had higher mean scores. Besides, there was a significant difference in mean score for the variable of work experience in Physical, Psychological and Social domains as well as general health. The healthcare staff having work experience of more than 15 years had higher mean scores.

These findings were similar to the results of a study by Alharbi and colleagues among nursing staff in Saudi hospitals where respondents who were 47 years or older were more satisfied (Alharbi et al., 2019). An explanation to this occurrence could be that the staff above the age of 40 years would have had more work experience and better understanding of nature of work and employment conditions. The HCPs may have developed many professional relationships in those years of work experience and may have had plenty of opportunities for further learning. In addition, they may have been able to achieve a better work-life balance and socio-economic status (Kaddourah et al., 2018, Alharbi et al., 2019).

There was a significant difference (p < 0.05) in mean score for the variable of gender in Psychological and Environment domains as well as general health. The male staff had higher mean scores. A possible reason for this occurrence could be the result of sampling as majority of respondents in our study was from nursing. It is mentioned in literature that nursing staff working in Saudi health sector have moderate or lower QWL (Almalki et al., 2012, Alharbi et al., 2019). Therefore, having a higher number of nursing staff who were usually females, as respondents in the study may have led to these results. Similarly, there was a significant difference (p < 0.05) in mean score for the variable of social status in Physical, Social and Environment domains as well as general health. Those who indicated their social status as married had higher mean scores for the said domains and general health. A study in healthcare staff in Pakistani health sector also reported that those who were married had higher scores for QoL (Iqbal, 2020). Another study among pharmacists in Pakistani healthcare setting also reported a higher stress level among pharmacists who were single (Madeeha et al., 2017). A possible explanation for this occurrence is that HCPs who are single may have to bear the burden of work stress alone while those who are married may be able to share it with their partners and have better coping ability. Such measures may reduce the stress and hence married HCPs may have better work-life balance. However, it has to be investigated.

For the variable of occupation, there was a significant difference in Environment domain as healthcare professionals who were physicians had higher mean score as opposed to all other HCPs when grouped together as non-physicians. This is a novel occurrence as QWL among physicians, pharmacists and other allied health staff in Saudi healthcare setting has not been reported before. Available data reports a moderate-to-lower QWL among nursing staff in Saudi hospitals (Almalki et al., 2012, Kaddourah et al., 2018, Alharbi et al., 2019). However, this presents an opportunity to further investigate the reasons for having a better QWL among physicians in Saudi health sector as compared to other professions.

There was a significant difference (p < 0.05) in mean score for the variable of extra working hours in Physical and Environment domains as well as overall QoL and general health. The staff who had no extra working hours had higher mean scores. A study in Saudi nursing staff mentioned unsuitable working hours as one of the reasons for dissatisfaction (Almalki et al., 2012). Hence, it was logical to have lower QWL among staff who have had extra work shifts during the pandemic.

There is a limitation of a slightly low sample count for the healthcare professionals working with COVID-19 patients in the study. Despite sending the survey electronically through the institution’s communication office, a small number of responses were received from this stratum. A possible reason could be the time constraint. The frontline workers who were eligible to participate in the study as per the criteria may not have had the time to respond to the survey. Nonetheless, the study was able to achieve a statistically acceptable sample size that gives enough weightage to its findings. The study did not analyze the QWL of healthcare staff based on nationality. As it was evident from the data that most of the respondents were non-Saudis, it would be interesting to see if the QWL differs among Saudi and non-Saudi staff. Further studies are recommended in this regard.

## 5 Conclusion

The QWL among healthcare staff during COVID-19 pandemic was low. Demographic factors were mainly the determinants for a higher QWL while the variable of extra working hours was a determinant of lower QWL. Based on our findings, no COVID-19 related variables were observed to significantly affect the quality of work life of the healthcare staff. It could be said that the factors that contributed to a lower QWL were similar to the ones reported previously. Hence, this occurrence present an opportunity to further improve the service as such factors have been repeatedly observed. Addressing these factors may improve the standard of patient care that continues to be the aim of every healthcare service provider.

## Declaration of Competing Interest

The authors declare that they have no known competing financial interests or personal relationships that could have appeared to influence the work reported in this paper.
